# Topography of the Corrugator Supercilii Muscle Relative to the Eyebrow and Its Clinical Application in Botulinum Toxin Injections

**DOI:** 10.3390/toxins17020085

**Published:** 2025-02-13

**Authors:** Hyun Jin Shin, You-Jin Choi, Kang-Jae Shin, Wu-Chul Song

**Affiliations:** 1 School of Medicine, Konkuk University, Seoul 05029, Republic of Korea; shineye@kuh.ac.kr (H.J.S.); cyj7797@kku.ac.kr (Y.-J.C.); 2 Department of Ophthalmology, Konkuk University Medical Center, Seoul 05029, Republic of Korea; 3 Research Institute of Medical Science, Konkuk University, Seoul 05030, Republic of Korea; 4 Institute of Biomedical Science & Technology, Konkuk University, Seoul 05030, Republic of Korea; 5 Department of Anatomy, Research Institute of Medical Science, Konkuk University School of Medicine, Seoul 27478, Republic of Korea; 6 Department of Anatomy and Cell Biology, Dong-A University College of Medicine, Busan 49315, Republic of Korea; shinkj@dau.ac.kr; 7 Department of Anatomy, Catholic Institute of Applied Anatomy, College of Medicine, Catholic University of Korea, Seoul 06591, Republic of Korea

**Keywords:** blepharospasm, corrugator supercilii muscles, botulinum toxin, superimposition, eyebrow

## Abstract

The purpose of this study was to elucidate the topography of the corrugator supercilii muscle (CSM) relative to the eyebrow with the aim of providing topographical guidance for botulinum toxin type A (BTX-A) injections in the East Asian population. Thirty-six hemifaces of 18 donated bodies for anatomical studies were dissected. Prior to dissection, four specific points on the eyebrow were marked to serve as reference points. A superimposition method for analyzing the position of the CSM relative to the eyebrow involved overlaying an image showing the dissected muscle onto a pre-existing image that contained reference lines indicating the eyebrow landmarks. The CSM almost overlaps the eyebrow at its medial end. Significantly, the central part of the CSM’s width was positioned just above the upper point of the eyebrow, being closely aligned with the midpupillary line. There was minimal overlap of the CSM beyond the midpupillary line on the lateral side, indicating that this muscle becomes relatively scarce or less distinct as it extends laterally from the midpupillary line. For effectively targeting the CSM, it is recommended to inject BTX-A precisely at the center of the medial end of the eyebrow just above the midpupillary line.

## 1. Introduction

The corrugator supercilii muscle (CSM) plays a crucial role in facial expressions by drawing the eyebrows medially and inferiorly to produce vertical wrinkles over the glabella and a frowning expression; it is often referred to as the “eyebrow muscle” [1]. Abnormal involuntary contractions of the CSM are clinically implicated in facial dystonia conditions such as blepharospasm [2]. Additionally, the CSM is associated with chronic migraine headaches caused by the entrapment of the supratrochlear nerve within the muscle [3,4].

Botulinum toxin type A (BTX-A) injections into the glabella area targeting the CSM have recently become popular for both cosmetic and therapeutic purposes [5]. It was reported that injecting 10 units of BTX-A (Botox, Allergan Ine, Irvine, Calif) effectively removed glabellar wrinkles in 80% of cases for up to 5 months [6,7]. In a therapeutic application, Yahalom et al. [8] demonstrated that injecting BTX-A into the CSM and the orbicularis oculi muscle resulted in moderate or marked improvement in 84.9% of cases. Blumenfeld reported reductions in headache frequency and intensity in 85.6% of 271 patients following BTX-A injections into the CSM and the procerus muscle [9]. Interestingly, randomized double-blind placebo-controlled trials have shown that BTX-A has antidepressant effects when it is injected into the frown musculature, including the CSM [10,11,12].

Knowledge of the topography of the CSM is vital for precisely administering BTX-A to ensure effective treatments. The spatial orientation of the CSM has been previously mapped using various anatomical markers, including the supraorbital rim and nasion [1,13,14]. However, these deep bony landmarks are not readily accessible in living subjects and are less intuitive than external landmarks, making their use less common in clinical practice [15,16]. In addition, clinicians predominantly rely on the position of the eyebrow rather than bony landmarks to guide BTX-A injections. However, there is a lack of information on the position of the CSM relative to the eyebrow. This gap in the literature highlights the need for more detailed studies to better understand the topographical relationship between the CSM and the eyebrow.

This study conducted an anatomical investigation to elucidate the topographical relationship between the CSM and the eyebrow with the aim of providing clinical guidance for BTX-A injections based on eyebrow landmarks, using donated bodies for anatomical research. This article also presents a method for superimposing the images of the eyebrow and the CSM that makes it possible to analyze regions in the glabella and supraciliary arch where the CSM overlaps the eyebrow [17,18]. This anatomical proposal may improve the efficacy of BTX-A injections in patients with blepharospasm and migraine, and it may also be beneficial in various esthetic procedures, including treatments for glabellar frown lines.

## 2. Results

Measurements related to the CSM and eyebrow landmarks were meticulously cataloged. Due to the variability in the position and shape of the eyebrow among individuals, careful attention was given to accurately identifying the positions of the four key landmarks (A1, A2, B1, and B2). The dissection revealed that the medial aspect of the CSMs was distinct and could be easily separated from the orbicularis oculi muscle by the intervening fascia. In contrast, the lateral aspect of the CSM beyond the midpupillary line often blended with the inferior part of the frontalis muscle, sometimes without a clear separating fascia.

**Figure 1:**
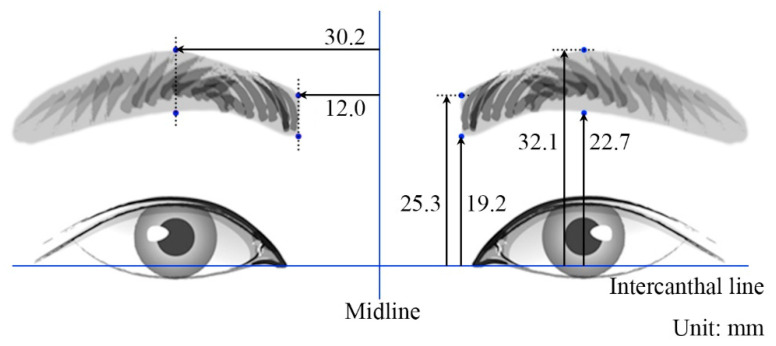
Mean measured distance values (refer to Figure 3 for the numbering scheme).

The measured distances are detailed in Table 1 and illustrated in Figure 1. The mean values from #1 to #6 were 30.2 mm, 12.0 mm, 25.3 mm, 19.2 mm, 32.1 mm, and 22.7 mm, respectively. There were no significant sex-related differences in these measurements (all *p* > 0.05).

**Figure 2:**
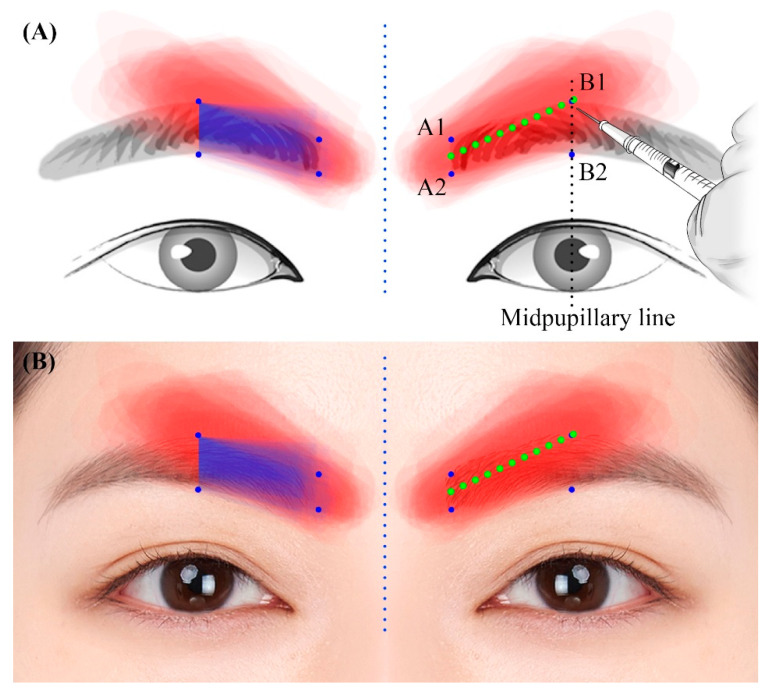
Superimposed schematic (**A**) and photograph (**B**) of the eyebrow and the CSM. Blue shading, overlapped eyebrows. Red shading, overlapped CSMs. Green dotted lines, recommended line along which to inject botulinum toxin type A into the CSM. Blue dotted line, midline.

Superimposing the images produced significant findings (Figure 2). The CSMs (red shading in Figure 2) were less evident, both above the upper point and below the lower point at the medial end of the eyebrow (blue shading in Figure 2). Furthermore, the middle section of the CSM’s width was located just above the upper point of the eyebrow, crossing the line through the center of the pupil (B1) (Figure 2A, right). The lateral part of the CSM was also relatively scarce beyond the midpupillary line.

## 3. Discussion

Botulinum toxin injections in the corrugator muscle are typically guided by the position of the eyebrow, but detailed information on the CSM’s precise location relative to the eyebrow is limited. This study aimed to enhance our understanding of the average dimensions of the CSM using identifiable landmarks to improve clinical applications. The medial eyebrow served as a key reference point, and an image superimposition technique was employed to analyze the CSM’s position. The results of the present study indicate that the CSM originates further inward than the medial end of the eyebrow and gradually disappears beyond the midpupillary line. The eyebrow and the CSM almost overlap at its medial end. However, as it extends outward, this muscle gradually rises above the lateral eyebrow, with its middle part located just above the upper corner of the eyebrow at the midpupillary line (Figure 2A, right).

Previous anatomical studies have focused on the location of the bony orbit, with few studies investigating the exact anatomy of the CSM with respect to the eyebrow. Characterizing the anatomical positional relationship between the eyebrow and the CSM has been challenging because they exist in two distinct layers, making a three-dimensional anatomical analysis necessary. Moreover, previous textbooks and research articles have described the optimal BTX-A injection point for the CSM as being located above the eyebrow or along the upper margin of the eyebrow irrespective of race [19,20]. However, our study revises and clarifies the previous anatomical understanding, finding that a large part of the CSM is located beneath the eyebrow in the East Asian population, except in the lateral parts where the CSM fibers are positioned superior to the eyebrow.

Considering the position of the CSM relative to the eyebrow, it is standard clinical practice for the injection points of BTX-A to include the middle of the eyebrow from the medial part to the upper point of the eyebrow at the midpupillary line (Figure 2A, green dotted line). Although there may be variations between patients, if BTX-A is injected at two points into the CSM [21], we recommend targeting the inner and outer endpoints of the green dotted line in Figure 2B. Not accounting for anatomical differences between Asians and Caucasians may reduce the efficacy of injecting BTX-A above the eyebrow in the medial part. The lateral point corresponds to the crossing point of the midpupillary line and the superciliary arch. Except in very special cases, we believe there is no need to inject BTX-A beyond the midpupillary line due to the scarcity of the CSM in this area and the mingling of the frontalis muscle with the CSM. If symptoms are severe and the effect does not last long enough, making an additional injection at the midpoint between the medial and lateral injection points along the green dotted line can be considered.

The CSM is thicker at the medial canthus than at the midpupillary line, which is an important consideration when injecting BTX-A [21,22]. The effect of BTX-A on the CSM is maximal, with deep injections made medially into the belly of the muscle near its origin at the point of its maximal thickness. For the medial injection, the needle should be advanced deeply until it touches the periosteum, then withdrawn 2–3 mm, followed by slowly injecting BTX-A into the muscle [23]. In contrast, injections at the midpupillary line should be performed more superficially because the CSM inserts into the skin at this location [19,23]. A subdermal injection is preferable for the lateral points, and to avoid creating “Angry Bird” eyebrows, the injection dose over the lateral brow should be reduced.

The results reported here could also be used in surgical management procedures such as CSM myectomy [3,4]. CSM resection surgery may benefit patients with chronic migraines that are unresponsive to other treatments. This can also be applied to treat severe glabellar lines and severe blepharospasm when repeated BTX-A injections have not provided sufficient or lasting relief [24]. The medial eyebrow is used as an external landmark in CSM myectomy, with a skin incision made above the medial eyebrow to minimize postoperative scarring [25]. Thus, the topographical information of the CMS relative to the eyebrow, as revealed by the present study, reduces undesirable postoperative outcomes such as asymmetries, recurrence of glabellar lines, and awkward forehead motion, and helps improve the learning curve for less experienced oculofacial surgeons [10].

Previous anatomical studies of the eyebrow position in the East Asian population have suggested that the mean medial eyebrow height (vertical distance from the medial canthus to the upper eyebrow margin) ranges from 21.2 mm to 26.9 mm [26,27]. Our data for distance #3 fall within this range, with a mean of 25.3 mm. Additionally, previous studies indicated that the lateral part of the CSM inserts into the dermis at 30 mm above the medial canthus and 16–35 mm from the midline.^21^ Our results are also consistent with these findings, showing that the crossing point of the upper margin of the eyebrow at the midpupillary line (where the lateral part of the CSM attaches to the dermal layer) is located 30 mm lateral to the facial midline and 32 mm above the medial canthus. However, it is well known that facial structure differs between Asians and Caucasians. Yang and Kim [14] suggested that the vertical height of the CSM is shorter in Korean compared to Caucasian cadavers. Kunjur et al. [28] also reported that the medial eyebrow height was shorter in Caucasians than in Asians. Therefore, future studies should apply anthropometric analyses to further evaluate race-related differences in the position of the CSM relative to the eyebrow.

In cases where the eyebrows are absent, such as when they are shaved or torn off, the eyebrow position can be predicted using alternative anatomical landmarks established in this study. Specifically, we utilized the facial midline and intercanthal distance as reference points to determine eyebrow positioning (Figure 1). The position of the canthus, as demonstrated in our previous studies, remains stable with age and serves as a reliable anatomical landmark [29]. By referencing these stable points and incorporating the data measured in this study, our findings can be effectively applied to guide botulinum toxin injections, even in the absence of eyebrows.

This study was subject to several limitations. We conducted this study using embalmed cadavers rather than fresh frozen ones. Formalin fixation typically results in approximately a 4% reduction in tissue width [30]. However, since embalming does not independently affect the relative positions of the eyebrow and CSM, we believe that their positional relationship would remain similar to that observed in fresh cadavers, and even in vivo. Most of the donated bodies for anatomical studies were elderly individuals. It is generally believed that the eyebrow position and shape change with age [31], and so the eyebrow position in elderly subjects might differ from that in younger individuals. Additionally, individual variation in eyebrow position must also be considered, as factors such as ethnicity and facial anatomy can contribute to differences in eyebrow positioning among individuals. However, previous studies have shown that the medial eyebrow is less mobile than the lateral eyebrow [27]. The lateral eyebrow is less supported by the frontalis muscle and so tends to descend in the aging population. In contrast, the morphology of the eyebrow is less variable in the medial part. Since the CSM is mainly present in the medial part of the eyebrow, the results of this study may be useful in clinical applications. In future studies, we plan to utilize 3D scanning technology to analyze additional dimensional parameters, such as the depth from the skin to the corrugator muscle and the muscle’s volume. Incorporating these factors will provide a more comprehensive understanding of facial anatomy and may enhance the precision of botulinum toxin injections.

## 4. Conclusions

In conclusion, due to the anatomical complexity and variability of the facial muscles, detailed knowledge of these muscles based on easily identifiable landmarks is essential for performing effective and safe procedures in the facial region. The topography of the CSM relative to the eyebrow shows that it is located beneath the eyebrow at the medial end, extends superior to the eyebrow, and is scarce beyond the midpupillary line in the East Asian population. This detailed topographical information about the CSM relative to the eyebrow may help less experienced surgeons achieve successful outcomes when performing various medical and surgical procedures.

## 5. Materials and Methods

Thirty-six hemifaces of 18 embalmed adult Korean donated bodies for anatomical studies were dissected. We obtained appropriate approval to use cadavers that had been legally donated to Dong-A University, Busan, South Korea (IRB No. 2-1040709-AB-N-01-202408-BR-005-02). The specimens included had no gross pathology or signs of surgical procedures in the upper eyelid, eyebrow, or forehead. After excluding 5 hemifaces that had been disrupted anatomically by previous dissection and trauma, 33 hemifaces were suitable for morphometric measurements, comprising 12 males and 6 females, and 15 right and 18 left sides. The mean age at the time of death was 76.9 years (range, 44–96 years). All procedures were performed in accordance with the Declaration of Helsinki of the World Medical Association. Each donor or their family had signed a document agreeing to participation in the body donation program of the medical school and the use of the body for clinical studies.

### 5.1. Anatomical Dissections and Measurements

**Figure 3:**
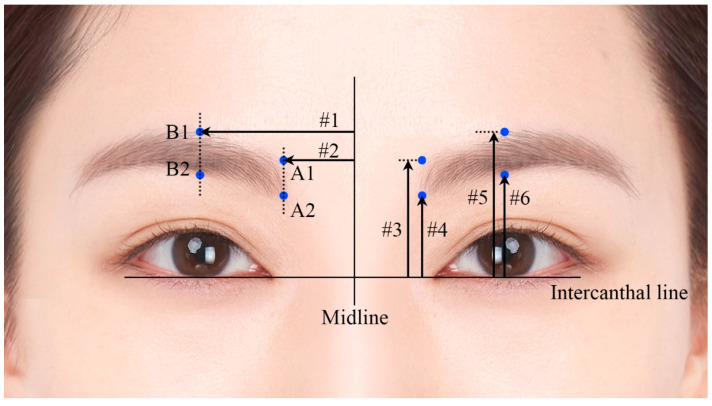
Landmarks and distances of the eyebrows for use as reference data. A1, upper point of the medial end of the eyebrow. A2, lower point of the medial end of the eyebrow. B1, upper point of the eyebrow crossing the midpupillary line. B2, lower point of the eyebrow crossing the midpupillary line. Distances #1 to #6 are explained in the Section 5 and their values are listed in Table 1.

The facial midline was used as a reference bony landmark, defined as a sagittal line running through the nasion (the junction between the frontal bone and two nasal bones) (Figure 3).

**Figure 4:**
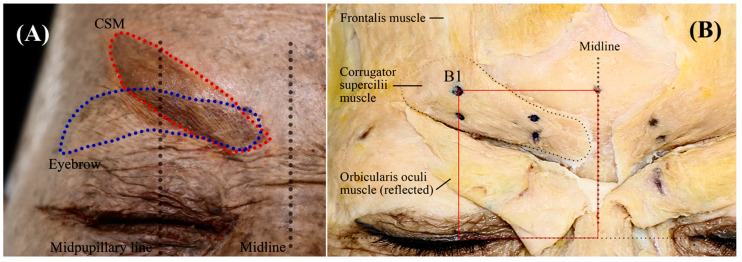
Superimposed image of the corrugator supercilii muscle (CSM) with the eyebrows (**A**) and anatomical details of the CSM (**B**). In panel B, the blue dots indicate eyebrow landmarks, while the red rectangle highlights the superimposed area.

An anatomical dissection demonstrating the location of the CSM is shown in Figure 4A,B. CSM is a small pyramidal muscle arising from the medial end of the supraciliary arch, inserting into the skin along the pupillary line, and covered by the orbital part of the orbicularis oculi and the frontal head of the occipitofrontal muscle. Anatomical dissections were performed by a transcutaneous approach in a layer-by-layer fashion. Four landmarks around the medial eyebrow were decided upon and deeply marked with pen from skin to bone before the dissection in order to ensure accurate superimposition and reference data, as follows (Figure 4B): the upper (A1) and lower (A2) points of the medial end of the eyebrow, and the upper (B1) and lower (B2) points of the eyebrow crossing the midpupillary line. After making these marks, photographs were taken with scale bars, and the following six distances around the eyebrow (designated as #1 to #6) were measured using Adobe Photoshop CS6 software (Adobe Systems, San Jose, CA, USA) with the accompanying scale bars, as follows (Figure 3):

#1. Facial midline to the midpupillary line (B1 or B2).
#2. Facial midline to the medial end of the eyebrow (A1 or A2).
#3. Intercanthal line to the upper point of the medial end of the eyebrow.
#4. Intercanthal line to the lower point of the medial end of the eyebrow.
#5. Intercanthal line to the upper point of the eyebrow crossing the midpupillary line.
#6. Intercanthal line to the lower point of the eyebrow crossing the midpupillary line.

After taking photographs, a meticulous dissection around the eyebrow was carried out. The skin was initially reflected downward, and the subcutaneous tissue covering muscles such as the frontalis, procerus, and orbicularis oculi muscles was removed. The clearly exposed orbicularis oculi, frontalis, and procerus muscles were then separated from the CSM and reflected downward. Finally, the CSM was clearly exposed by removing the remaining fascia. After exposing the CSM, the four eyebrow landmarks (A1, A2, B1, and B2) were confirmed once again and marked on the CSM with blue dots (Figure 4B). Additional photographs were then taken to perform the superimposition.

### 5.2. Superimposition

The superimposition process was performed using a standardized method that we have described previously [17,18]. We overlaid an image of exposed CSMs on top of an already existing image. The reference area for superimposition was created based on the measured reference distance. Before performing the alignment process, all left photographs of the samples were horizontally flipped to the right using Photoshop CS6 to allow alignment with the midline. The dissected images were imported into Photoshop CS6 and aligned to the midline, intercanthal line, and landmark B1 (Figure 4B, red rectangle). The CSMs and eyebrows connecting the four landmarks (A1, A2, B1, and B2) were highlighted using red and blue shading, respectively. Finally, we superimposed the image on top of an already existing image with reference lines and then added the subsequent images using Photoshop CS6.

### 5.3. Statistical Analyses

The mean and standard-deviation values of the variables were calculated using standard statistics software (version 27.0, SPSS for Windows, SPSS, Chicago, IL, USA). Sex-related differences in each parameter were analyzed statistically using an independent sample t-test, with the criterion for statistical significance set to *p* < 0.05.

## Figures and Tables

**Table 1:** Measurement items. Detailed descriptions according to number are provided in the text (Section 5 and Section 5.1) and Figure 3.

Anatomic Variables		#	Mean	SD	Range
Horizontal distance from facial midline	To midpupillary line	(1)	30.2	2.3	27.4~34.2
	To medial end of eyebrow	(2)	12.0	2.2	8.3~14.4
Vertical distance from intercanthal line	To upper point of medial end of eyebrow	(3)	25.3	3.8	16.1~30.5
	To lower point of medial end of eyebrow	(4)	19.2	2.0	16.1~22.6
	To upper point of eyebrow crossing midpupillary line	(5)	32.1	3.4	28.1~39.5
	To lower point of eyebrow crossing midpupillary line	(6)	22.7	3.1	17.6~30.5

## Data Availability

The original contributions presented in this study are included in the article. Further inquiries can be directed to the corresponding author(s).
